# Multi-Criteria Selection of Adhesives for Wearable Textiles

**DOI:** 10.3390/polym18121504

**Published:** 2026-06-16

**Authors:** Bhalaji Yadav Kantepalle, Udena Epitawala Arachchige, Daeha Joung, Christina Tang

**Affiliations:** 1 Chemical and Life Science Engineering, Virginia Commonwealth University, Richmond, VA 23284, USA; 2 Department of Physics, College of Humanities and Sciences, Virginia Commonwealth University, Richmond, VA 23284, USA; epitawalaaru@vcu.edu (U.E.A.); joungd2@vcu.edu (D.J.)

**Keywords:** wearable textiles, soft substrates, commercial adhesives, T-peel test, stick–slip, fracture energy, IC-Peel, pressure-sensitive adhesives

## Abstract

Peeling behavior of soft materials is important in a wide range of applications, e.g., electronics, healthcare, etc. When applied on soft substrates, soft adhesives demonstrate unique mechanical behaviors compared to adhesives applied on rigid substrates. Adhesive properties can be conveniently measured by “peel testing”. The focus of this work is characterization of commercial glues on fabric substrates using commonly used peel tests. We investigate energy dissipation on textile substrates. For practical applications, we aim to develop a systematic approach for selecting adhesives for soft, flexible substrates. Here, we developed a multi-criteria framework for evaluating adhesives using data from peel tests. The criteria used here consider the shape and stability of the T-peel trace. The results of the multi-criteria evaluation were compared to traditionally used peel strength and fracture energy. Although E6000 produced the highest peel force ($1.82\pm 0.27\;N\;mm^{-1}$) and the largest apparent fracture energy, $G_{c}$ ($8673\pm 1545\;J\;m^{-2}$), it showed large force oscillation (SSA=4.05±0.83 N). Fabri-Fuse was selected based on its low oscillation (SSA=0.69±0.29 N), lowest $CoV_{F_{ci}}$(4.0%), high peel stability index (PSI), and high displacement at break. Functional evaluation showed that Fabri-Fuse increased strain-to-electrical-failure to 34.95±2.43%, higher than direct printing on fabric or printing on E6000 (highest peel strength). These results suggest that metrics that consider the shape of the peel trace and inter-sample repeatability provide a useful alternative for selecting adhesives other than highest peel strength.

## 1. Introduction

Understanding peeling behavior in soft materials is important in a wide range of applications, e.g., electronics, healthcare, packaging, etc. Soft adhesives, especially when applied on soft substrates, demonstrate unique mechanical behaviors compared to adhesives applied on rigid substrates [1]. The adhesive properties of adhesives on stretchable substrates [2] and multilayer systems [3] can be conveniently measured by “peel testing”. Standard methods (e.g., ASTM) exist for measuring peel strength. However, the results of the tests are strongly dependent on test geometry, sample thickness, peeling rate, environmental testing conditions (temperature and moisture), and plastic deformation in the peel arm(s) [2,4]. Thus, using the common standards, the peel strength cannot always be considered a “material property” or “fundamental property”. Furthermore, the specific aspects of the test interpretation can be complex. This complexity has led to significant challenges across the fields that use peeling, where poor measurement methods and interpretations can lead to incorrectly reported or misleading material properties [4].

Debonding of a peel joint can be considered a cracking process in which a crack initiates and propagates as the joined materials separate [4]. The analysis of this process can provide the fracture energy (critical energy release rate in units of energy per unit area, $G_{c}$) [4]. Fracture energy can be calculated from peel test data using the energy analysis approach [4]. The principle of energy conservation is fundamental to adhesive systems, and the analysis framework is universal [1]. Ideally, fracture energy can approach the thermodynamic work of adhesion [4]. The measured $G_{c}$ can be 1000-fold greater (for tough engineering adhesives) [4]. Additionally, peel tests in which the peel arm (i.e., substrate) is flexible can result in considerable energy dissipation, several orders of magnitude larger than the energy for debonding [4].

Fracture energy, energy dissipations, and “correction parameters” have been investigated for polymer films [3] and stretchable plastic films [2]. In this work, we investigated fracture energy (the sum of adhesive fracture energy and plastic work in bending) on fabric substrates. We investigated energy dissipations of fabric substrates. In particular, we were interested in investigating bending and tensile corrections of the peel of fabrics compared to polymer films reported in the literature and determining if the type of glue affected the energy dissipation of the peel arm for various classes of commercial pressure-sensitive adhesive glues.

For practical applications, several system-specific approaches to peel test design and analysis have been reported. For example, peel-force histories to quantify force fluctuations and uncertainty [5], coupled parametric/statistical protocols have been used to reduce variation and improve repeatability in quantitative thin-film peel measurements [6], cassette-like and fixture-assisted peel systems have been developed to improve control of the peeling front or reduce parasitic loading in soft-to-rigid and thin flexible laminate assemblies [7], and adapted adhesion tests have been used for polymer nanofiber–fabric interfaces [8]. This prior work demonstrates that peel-force histories contain valuable information complementary to a single average value, or peel strength. However, these approaches can require specialized testing setups.

The focus of this work is characterization of commercial glues on fabric substrates using commonly used peel tests. We investigate energy dissipation of fabric substrates. For practical applications, we aim to develop a systematic approach for selecting adhesives for soft, flexible substrates using peel testing. We develop a multi-criteria method for evaluating adhesives on soft, flexible substrates using data from a standard T-peel test. Displacement at break $\left(x_{break}\right)$ was used as a measure of how much extension each bonded fabric assembly accommodated before failure. Additional criteria, including the peel stability index (PSI), stick–slip amplitude (SSA), and crack-initiation-force coefficient of variation $\left(CoV_{F_{ci}}\right)$, were defined to consider the shape and stability of the peel trace. Using these performance-based metrics, we aim to establish a proof-of-concept for adhesive selection on fabric substrates as an alternative to peel strength alone. These combined criteria were compared to the traditionally used peel strength and fracture energy. Adhesives were selected based on the multi-criteria method, and the functional performance of the selected adhesive was evaluated by measuring its effect on strain-to-electrical-failure for a printed conductive path.

## 2. Materials and Methods

### 2.1. Materials

Knit fabric (82% nylon and 18% spandex, 187.15 ± 0.61 gsm (n= 3), Performance Black SD Solid, JOANN Fabrics & Crafts, Richmond, VA, USA) was used as a model fabric substrate. Four commercial adhesives were evaluated for fabric–fabric bonding: E6000 Industrial Strength Adhesive, Fabri-Fuse Adhesive, JB Weld 31310 All-Purpose RTV Silicone, and Guo Elephant 407 instant adhesive. For functional assembly studies, conductive traces were 3D printed using a flexible silver conductive ink (AA-DUCT 916 Flexible Silver Epoxy, Atom Adhesives, Providence, RI, USA). All materials were used as received.

### 2.2. Adhesion Strength Characterization

The fabric was ironed and cut into 40 mm × 50 mm pieces. All peel specimens were cut parallel to the wale direction (40 mm edge in wale direction). Fabric–fabric T-peel laminates were prepared by applying approximately 300–400 mg of adhesive to the technical back of one adherend, aligning a second adherend in the same fabric direction, and allowing the assembly to cure under adhesive-specific handling conditions (schematic in Figure S1). For E6000, Fabri-Fuse, and JB Weld, the adhesive was spread across the intended bonded region before lamination; for Guo Elephant 407, direct application during clamping was used because of its rapid setting behavior. The adhesives and curing conditions used are summarized in Table S1. Because the peel response can be affected by adhesive-layer thickness and bonded-area geometry, adhesive application mass, overlap dimensions, and specimen geometry were controlled as consistently as practicable during laminate preparation [9,10]. After curing, each parent laminate was center-cut to obtain final 20 mm× 50 mm T-peel specimens containing a 20 mm× 25 mm bonded overlap and unbonded ends for gripping. Adhesive-specific curing details are summarized in Table S1.

Peel testing was performed in T-peel geometry using an adapted apparel-fabric protocol based on ASTM D2724-19 [11,12]. Briefly, fabric–fabric specimens measured 20 mm × 50 mm with a 20 mm × 25 mm bonded overlap. Tests were conducted with opposing grips, a 20 mm initial clamp gap, and a crosshead speed of $10\;mm\;{min}^{-1}$($0.17\;mm\;s^{-1}$). T-peel tests were performed using a texture analyzer (TA.XTPlus Connect, Stable Micro Systems, Godalming, Surrey, UK). The samples were prepared so that the direction of peel was parallel to the wale direction of the sample (Figure S1). This was consistent in all samples to ensure that all adhesive systems were compared under the same fabric orientation. The fabric orientation has been reported to affect peel strength. Specifically, higher peel strengths have been reported when the wale direction was perpendicular to the peel direction [13]. The difference has been attributed (in part) to differences in contact splitting mechanics (i.e., contact that inhibits crack propagation) [13]. Notably, fiber content, knit structure, and fiber morphology may also affect adhesion. Future work further investigating the effect of textile properties on peel behavior is of interest. In this work, we investigated a single fabric orientation (likely representing the orientation of lower performance).

All experiments were conducted under ambient laboratory conditions of 19–22 °C and 26–52% relative humidity (RH). At least three replicate specimens were tested for each adhesive/substrate condition. Following the peel test, both sides of the sample were imaged as a preliminary evaluation of fracture [14,15].

### 2.3. Data Analysis

Raw force–displacement data were exported and processed in Python 3.12.13. Each trace was baseline corrected. Sliding 25 mm displacement analysis windows were evaluated across each baseline-corrected force trace beginning 5 mm after displacement onset. Candidate windows were required to contain at least 10 data points, remain outside the terminal 20% of the recorded displacement range, and satisfy a slope/drift filter to ensure selection of a stable plateau. Among the possible windows, the window with the lowest sample standard deviation in force was used for further analysis. The stable-window selection, extrema-extraction procedure, and detailed peak-detection settings are illustrated in Figure S2.

Within the selected window, the five highest local maxima and five lowest local minima were identified. $F_{c}$ was computed as the average of the retained five highest local maxima and five lowest local minima:

(1) $$F_{c}(N)=\frac{\sum_{i=1}^{5}F_{p,i}+\sum_{j=1}^{5}F_{v,j}}{10},$$

where $F_{p,i}$ is retained local peak force i, and $F_{v,j}$ is retained local valley force j. The resulting $F_{c}/w$ $\left(N\;mm^{-1}\right)$ value followed the ASTM D2724-19 [11] convention of reporting peel/bond strength normalized by specimen width. Local maxima and minima were identified directly from the baseline-corrected force trace within the selected window without additional smoothing. The peel strength, $F_{c}/w$, was calculated as a traditional performance metric.

In this work, we defined additional criteria to screen and evaluate adhesives for soft, flexible substrates. Crack-initiation force, $F_{ci}$, was defined as the mean of the retained five highest local maxima:

(2) $$F_{ci}(N)=\frac{\sum_{i=1}^{5}F_{p,i}}{5}\;.$$

Additional criteria considering the shape and stability of the peel trace were defined. Specifically, the peel stability index (PSI) was defined as the ratio of mean window force to sample force standard deviation:

(3) $$PSI(\frac{N}{N})=\frac{{\bar{F}}_{win}(N)}{s_{F,win}(N)},$$

where ${\bar{F}}_{win}$ and $s_{F,win}$ are the sample mean and sample standard deviation, respectively, of the baseline-corrected force values within the selected analysis window. Stick–slip amplitude (SSA) was defined as the amplitude difference between the mean of the retained five local maxima and the mean of the retained five local minima:

(4) $$SSA(N)={\bar{F}}_{p}-{\bar{F}}_{v},$$

where ${\bar{F}}_{p}$ and ${\bar{F}}_{v}\;$ are the mean local maxima and the mean of the local minima, respectively. Thus, SSA was considered a measure of the shape of the peel trace rather than a material property. Inter-sample repeatability was quantified using the coefficient of variation of $F_{ci}$:

(5) $$CoV_{F_{ci}}(\%)=100\frac{s_{F_{ci}}}{{\bar{F}}_{ci}},$$

where $s_{F_{ci}}$ and ${\bar{F}}_{ci}$ are the sample standard deviation and mean of $F_{ci}$ across replicate specimens.

ChatGPT (OpenAI, GPT-5.2 through GPT-5.5 Thinking models; accessed December 2026–May 2026) was used to assist with Python code development, debugging, and documentation. All code outputs, extracted values, calculations, and figures were reviewed and verified by the authors.

### 2.4. Peel Fracture Analysis

For comparison with the tensile-derived substrate yield reference, crack-initiation force was also converted to nominal peel-arm axial stress:

(6) $$\sigma_{ci}\left(MPa\right)=\frac{F_{ci}}{wt},$$

where w and t are the specimen width and peel-arm thickness, respectively. 

Peel fracture analysis was performed using the Imperial College (IC)-Peel procedure (IC-Peel 2006 version). To obtain substrate mechanical properties required for subsequent peel fracture analysis, uniaxial tensile tests were performed on the same black 82% nylon/18% spandex fabric used in the peel experiments, with specimens cut in the wale direction to match the primary peel specimen orientation. Each tensile specimen had a width of 50 mm, an overall length of 150 mm, a thickness of approximately 0.40 mm, and an effective gauge length of 75 mm, and was tested in force–displacement mode at a crosshead speed of $300\;\pm \;10\;mm\;{min}^{-1}$ ($12\;\pm \;0.5\;in\;{min}^{-1}$). Force–displacement data were converted to engineering strain and nominal stress using the measured specimen dimensions and were processed in Python 3.12.13. Following toe compensation, the elastic modulus, E, was estimated from the 0.02–0.08 strain (ε) window using a slope-through-origin fit. The data ε ≥ 0.06 were fit using a linear-plus-power-law model. For each specimen, the E, $\sigma_{y}$, $\varepsilon_{y}$, and the power-law hardening exponent, $n_{PL}$, were determined (n= 3).

Substrate properties (E, yield stress $\sigma_{y}$, yield strain $\varepsilon_{y}$, and power-law hardening exponent $n_{PL}$) were then combined with the peel-force input, $F_{c}$, in the IC-Peel procedure to compute peel fracture energy outputs [16]. Additional details of the tensile-to-IC-Peel input-extraction workflow, including toe/slack removal, elastic-window fitting, post-knee constitutive fitting, model selection criteria, and exported IC-Peel inputs, are described in the Supplementary Material. The inputs used for IC-Peel, including fabric substrate elastic modulus E, yield stress $\sigma_{y}$, yield strain $\varepsilon_{y}$, power-law hardening exponent $n_{PL}$, fabric thickness h, sample width b_peel_, and adhesive-specific $F_{c}$, are reported in Table S2, and θ = 180° was used as the T-peel-equivalent high-angle input, as described in the Supplementary Material.

### 2.5. Functional Evaluation

To evaluate the functional performance of the adhesive, static shear testing was performed on folded-loop fabric specimens prepared with a 10 mm × 20 mm lap area. The bonded specimens were clamped vertically and loaded incrementally until adhesive debonding occurred, and the static shear failure load, $F_{s}$, was recorded in newtons (N).

To mimic a wearable device, conductive traces were printed on the textile substrates using a flexible silver conductive ink (AA-DUCT 916 Flexible Silver Epoxy, Atom Adhesives, Providence, RI, USA) by direct ink writing (DIW) on a custom robotic gantry system (A351, Physik Instrumente L.P., Karlsruhe, Germany) equipped with a pneumatic dispensing unit (Ultimus V, Nordson EFD, Westlake, OH, USA). Ink was extruded through a metal nozzle to produce straight conductive lines of dimensions 30 mm (in length) × 0.5 mm (in width). To evaluate the role of the adhesive layer, three substrate conditions were investigated: textile without adhesive, textile freshly coated with Fabri-Fuse, and textile freshly coated with E6000. Rectangular fabric substrates measuring 60 mm × 15 mm were used to compare printed conductive features prepared with and without an adhesive layer. For the no-adhesive control, the silver conductive trace was printed directly onto the fabric substrate. For the adhesive-assisted conditions, a thin layer of Fabri-Fuse or E6000 was applied immediately before printing, and the conductive ink was then printed directly onto the uncured adhesive layer. After printing, the samples were cured for 48 h at ambient conditions.

The prepared specimens were evaluated under uniaxial extension using a texture analyzer (TA.XTPlus Connect, Stable Micro Systems, Godalming, Surrey, UK). Samples were mounted and stretched at a constant rate of 0.17 mm s^−1^, while the electrical response of the printed conductive path was monitored simultaneously using a source meter (2450 SMU, Keithley, Keithley Instruments, Solon, OH, USA). A constant applied voltage, $V_{app}$, of 1 mV was applied across the printed trace, and the resulting current, I, was recorded continuously during deformation. Electrical failure was defined as the point at which the measured current dropped abruptly to zero, indicating loss of electrical continuity in the conductive path. Strain-to-failure was calculated as 100 times the extension at electrical failure divided by the initial mounted gauge length, $L_{0}$ = 40 mm, and was reported as a percentage. Three replicate specimens were tested for each condition (n= 3). The resulting strain-to-failure values were used to compare device-level deformation tolerance across the no-adhesive control and adhesive-assisted conditions.

## 3. Results and Discussion

### 3.1. Failure Analysis

Adhesives are widely used, but no single adhesive is suitable for all applications. Selecting the appropriate adhesive for a given application is important for achieving reliable bonding because adhesive performance depends on multiple interrelated factors, including substrate type, joint geometry, loading mode, processing conditions, and service environment [17,18]. The goal of this work was to develop a systematic approach for selecting adhesives for soft, flexible substrates using peel testing, a commonly used method for evaluating bonded flexible materials [4,11,19]. As a model fabric substrate, we selected 82% nylon/18% spandex weft knit (jersey) fabric. Nylon/spandex knit blends have been previously used for e-textiles and weft knits are versatile for general apparel applications [20]. To demonstrate this approach, four commercial adhesives were considered for fabric-to-fabric bonding on the 82% nylon/18% spandex knit textile substrate: E6000 Industrial Strength Adhesive, Fabri-Fuse Adhesive, JB Weld 31310 All-Purpose RTV Silicone, and Guo Elephant 407 instant adhesive. Different classes of pressure-sensitive adhesives were investigated (polyurethane-based (E6000), rubber-based (Fabri-Fuse), and RTV silicone (JB Weld)). We compared pressure-sensitive adhesives to a model structural adhesive (cyanoacrylate (Elephant 407)). Information about the commercial glues used in this work is summarized in Table S1.

T-peel tests of fabric–adhesive–fabric samples were performed. As an initial analysis of failure, the samples were visually inspected following the peel test. Fracture surface analysis based on images has been previously reported [15]. Images of the surfaces after the peel test are shown in Figure S3. Based on the visual analysis of the surface, the sample was classified as adhesive, cohesive, or mixed failure (no substrate failure was observed). Cohesive failure was assigned when adhesive residue remained on both separated fabric surfaces. Adhesive failure was assigned when one fabric surface was largely clean and the adhesive remained primarily on the opposing adherend [4,21]. Mixed failure was assigned when residue distribution varied along the bonded region or when local fabric disturbance accompanied adhesive separation. Fabri-Fuse showed a comparatively uniform post-peel surface appearance, consistent with a distributed apparent mixed/cohesive failure mode. E6000 showed larger light-colored residue regions and stronger contrast between opposing separated surfaces, consistent with apparent mixed failure involving substantial residue transfer and local textile deformation. JB Weld RTV silicone showed patchier residue on the separated textile surfaces, consistent with an apparent mixed/cohesive residue pattern. In contrast, Elephant 407 showed more localized and irregular residue, consistent with an apparent mixed/interfacial failure appearance. These results are consistent with previous observations for structural adhesives (Elephant glue) [22] and pressure-sensitive adhesives [23]. Because these assignments are based on visual inspection of specimen-scale residue patterns, we treat them as qualitative failure-surface observations rather than analysis of fracture patterns. Analysis of microscopic mechanisms (e.g., cleavage fracture and silver patterning) is of interest in future work [23,24,25].

### 3.2. Peel Strength and Fracture Energy Analysis

**Figure 1:**
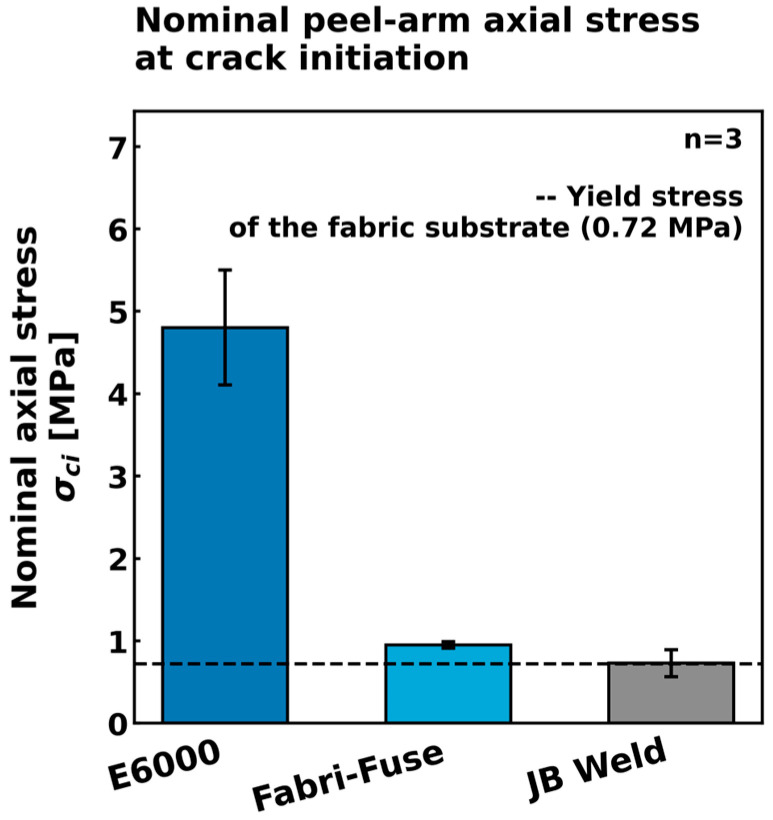
Nominal peel-arm axial stress compared to the yield stress of the substrate. Bars show mean ± SD (n = 3). The dashed line indicates the yield stress of the fabric substrate.

Representative force–displacement traces from the T-peel tests are shown in Figure S2. Based on the T-peel traces, peel/bond strength normalized by specimen width was calculated, with detailed values provided in the Supporting Information. We confirmed our force-trace analysis methodology using Scotch Magic™ Tape 810 (3M, Maplewood, MN, USA) as a reference. The peel strength of Scotch 810-to-Scotch 810 T-peel was $F_{c}/w\;=\;0.187\;\pm \;0.024\;N\;mm^{-1}$, which was consistent with reported Scotch 810 autohesion values [26] (details about sample preparation are reported in the Supplementary Material, Table S3. For the fabric–adhesive–fabric samples, E6000 showed the highest peel/bond strength, followed by Fabri-Fuse and JB Weld (Table 1). The peel strength of Elephant 407 was not determined because no stable plateau in the force was observed. We also estimated the axial stress in the peel arm. Crack-initiation force was converted to nominal peel-arm axial stress using sample width and approximate thickness. The resulting $\sigma_{ci}$ values were compared to the yield stress of the fabric substrate (0.723 ± 0.343 MPa; Figure 1).

E6000 showed the highest crack-initiation force (38.43±5.59 N) and the highest corresponding nominal peel-arm axial stress (4.80±0.70 MPa), substantially exceeding the tensile-derived substrate yield reference. Fabri-Fuse and JB Weld showed lower crack-initiation forces (7.62±0.31 N and 5.84±1.30 N, respectively), corresponding to lower nominal peel-arm stresses of 0.95±0.04 MPa and 0.73±0.16 MPa, respectively. Initiation stresses, $\sigma_{ci}$, exceeding the yield stress of the substrate ($\sigma_{y}$) may indicate peel-arm deformation and dissipation [1,4].

Notably, these results of peel strength alone can be misleading and include peel-arm deformation and associated energy loss [1,4,7]. IC-Peel 2006 software has been developed by Imperial College to automate the energy balance calculations involved with creating surface as part of a peel test. The calculations involve numerical integration of the bilinear fit of experimental stress–strain data and require the Young’s modulus of the substrate, the thickness of the substrate, the yield stress of the substrate, and the peel geometry [16,27]. The principle of energy conservation is fundamental to adhesive systems, and the framework is universally applicable for adhesive structures [1]. This approach has previously been applied to soft substrates, including stretch wrap (LDPE film) [2]; thus, in this work we have applied this approach to textile substrates. Our objective is to investigate energy dissipation on textile substrates. In particular, we were interested in investigating bending and tensile corrections of the peel of fabrics compared to polymer films reported in the literature and determining if the type of glue affected the energy dissipation.

IC-Peel software automates numerical integration of the bilinear fit of experimental stress–strain data based the Young’s modulus of the substrate, the thickness of the substrate, the yield stress of the substrate, and the peel geometry. According to Moore and Williams [16], solutions have been formulated for bilinear, power-law, or digitized stress–strain behavior. In this work, we used tensile testing data of the fabric 82% nylon/18% spandex knit as input for the IC-Peel analysis. The tensile force–displacement data were converted to nominal stress–engineering strain, toe/slack was removed, the early modulus was extracted from the selected low-strain window, and the post-knee response was fitted using bilinear and power-law forms. The power-law fit better captured the measured strain-stiffening response and was, therefore, used to define the primary IC-Peel inputs. The results using the bilinear fit have been included in the Supplementary Material for comparison (Table S2).

Using the power-law fit, the IC-Peel software was used to compute the apparent critical peel energy ($G_{c}$), dissipation energy, total energy, correction parameter, and maximum stress. E6000 had the highest computed $G_{c}$, followed by Fabri-Fuse and JB Weld. The order of magnitude of the fracture energies was $\sim 1000\;J\;m^{-2}$, as expected for pressure-sensitive adhesives [4]. The trend in maximum stress was comparable to axial stress calculations presented in Figure 1. Comparable to previous reports, bending of the peel dominated energy dissipation compared to tensile corrections [3]. From the IC-Peel test protocol, the plastic work in bending ($G_{d}$) was calculated and found the be much less ($\sim 1\;J\;m^{-2}$) and decreased slightly with decreasing adhesive toughness. This result is consistent with previous studies demonstrating that $G_{d}$ is affected by the adhesive type [2]. The low plastic work in bending was much lower than the adhesive fracture energy, resulting in low “correction” parameters (<0.1%; Tables S2 and S4). Notably, these “correction” parameters are significantly lower than those previously reported for analysis of adhesives on stretchable substrates [2] and multilayer systems [3]. Despite these apparent low correction parameters, energy dissipation in the peel arm was still possible, classified by IC-Peel in the elastic–plastic loading/unloading regime [28]. Therefore, the calculated apparent fracture energy may include peel-arm deformation and dissipation in addition to interfacial separation. $G_{d}$ represents plastic work associated with bending in the peel model [3], and fabric deformation may include deformation by, e.g., yarn sliding and rotations [29], rather than purely bending that is not captured by $G_{d}$. These results provide fundamental insight into energy dissipation of the fabric substrate during peel tests and are helpful for interpreting peel tests on fabric (e.g., coatings, wearable devices, etc.).

### 3.3. Adhesive Selection Beyond Peel Strength

**Figure 2:**
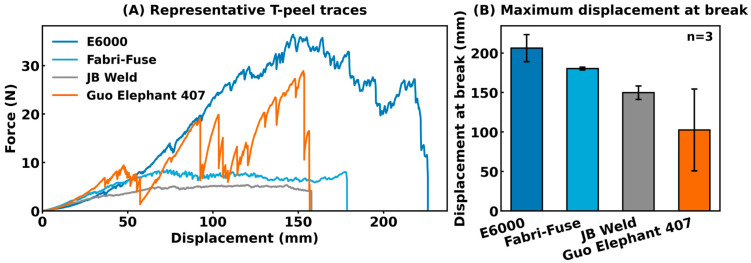
(**A**) Representative force–displacement curves showing differences in force level, peel-trace shape, and oscillatory behavior. (**B**) Maximum displacement at break for each adhesive, reported as mean ± SD (n = 3).

For practical applications, it is unclear if selecting the adhesive with the highest peel strength or fracture energy is the best option. Based on the peel tests, we observed that representative T-peel curves for the different adhesives showed substantial differences in force–displacement behavior. In particular, the traces differed not only in overall force scale but also in smoothness, oscillatory character, and the amount of displacement sustained before debonding. For example, Fabri-Fuse and JB Weld showed visibly steadier peel traces than E6000 (Figure 2A). Thus, the goal of this work was to develop a systematic approach for selecting adhesives for soft, flexible substrates based on analysis of the force–displacement traces from the T-peel tests.

The effect of the adhesive layer on fabric flexibility and the ability to remain attached over substantial deformation was examined by measuring maximum displacement at break (Figure 2B). Specifically, displacement at break reflects how much deformation the bonded system can tolerate before loss of load-bearing attachment. Figure 2B shows that maximum displacement at break further separated the adhesives by deformation tolerance, with E6000 reaching the largest mean displacement at break (206.2 ± 17.2 mm), followed by Fabri-Fuse (180.3 ± 1.8 mm), JB Weld (149.8 ± 8.5 mm), and Elephant 407 (102.5 ± 51.9 mm). Although E6000 showed the highest mean displacement at break, Fabri-Fuse remained comparably extensible while also exhibiting steadier peel behavior in the subsequent screening steps. By contrast, Fabri-Fuse sustained about 20% greater displacement before failure than JB Weld and about 76% greater displacement than Elephant 407. The poor deformation tolerance of Elephant 407 was attributed to the cyanoacrylate adhesive soaking into the fabric and causing the bonded region to become brittle. Thus, Elephant 407 was not further analyzed in the stability-qualified or energy-based comparison. The model liquid-based structural adhesive was not appropriate for fabric substrates.

We observed a direct correlation between displacement at break and peel strength (Figure S4). Peel tests on compliant adherends can include peel-arm deformation and viscoelastic/plastic dissipation in addition to interfacial separation [1,4,7]. Therefore, under the same textile T-peel geometry, adhesive systems that appear to sustain higher peel forces (i.e., high peel strength) can also remain load bearing over a longer displacement before failure occurs.

To better understand the effect of peel-trace shape beyond peel strength alone, criteria quantifying the force-trace shape, oscillation severity, and initiation-force (i.e., peak) repeatability were considered. Specifically, the peel stability index (PSI; Equation (3)) was used as a measure of stability, where a higher value indicates a lower oscillation in force response in the selected peel region.

**Figure 3:**
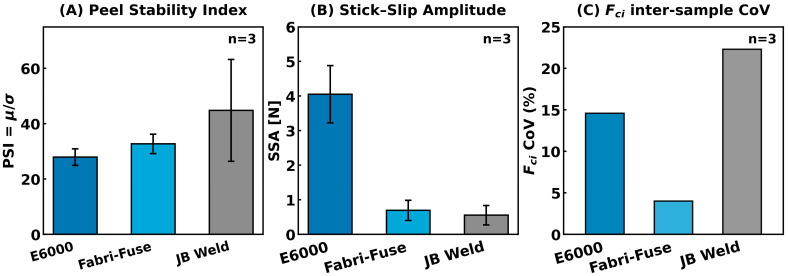
(**A**) Peel stability index (PSI; higher PSI indicates lower force oscillation), (**B**) stick–slip amplitude (lower SSA indicates lower force oscillation), and (**C**) peak force repeatability (inter-sample; lower $CoV_{Fci}$ indicates better reproducibility) for pressure-sensitive adhesive systems. Values are reported for n=3.

Comparing adhesives in terms of PSI, Fabri-Fuse (32.7 ± 3.5) and JB Weld (44.8 ± 18.4) showed a higher PSI than E6000 (28.0 ± 3.0; Figure 3A), corresponding to lower oscillations in the force response in the selected peel region. Relative to E6000, the mean PSI was approximately 17% higher for Fabri-Fuse and approximately 60% higher for JB Weld. Taken together, these results demonstrate that adhesives with the highest peel-strength response are not necessarily the adhesives with the highest peel stability.

Because PSI (Figure 3A) alone does not quantify the absolute size of force oscillations or the repeatability of force initiation across specimens, further criteria were considered. Stick–slip amplitude (SSA; Equation (4); Figure 3B) and the coefficient of variation of crack-initiation force, $CoV_{F_{ci}}\;(Equation\;\left(5\right);\;$Figure 3C), were used as complementary criteria to describe within-trace force oscillation and specimen-to-specimen repeatability, respectively. SSA  quantified the magnitude of force fluctuation (local maxima to local minima) within the selected peel window using the same local maxima and local minima used to calculate $F_{c}$. The $CoV_{F_{ci}}$ quantified the repeatability across replicate specimens (peak force). Low SSA was considered desirable because it indicated lower oscillation in force response in the selected peel region. Low $CoV_{F_{ci}}$ was considered desirable because it indicated more repeatable results under the same test conditions.

We also observed a direct correlation between SSA and peel strength (Figure S4). Conceptually, this direct correlation is not necessarily intuitive (i.e., larger slip can conceptually be associated with lower peel strength). We note that SSA is defined as the absolute force difference between local maxima and minima in the selected peel window. These local maxima and minima correspond to ($F_{ci}$)-like crack initiation/reinitiation force levels and ($F_{ca}$)-like post-slip or arrest force levels; thus, the larger the peel strength, the larger the magnitude of the crack initiation/reinitiation forces [4]. We observed no correlation between PSI or $CoV_{F_{ci}}$ and peel strength (R^2^ = 0.01, Figure S4). PSI is a statistical measure of the noise in the peel force trace (standard deviation/mean). Physically, no relationship between the mean and the standard deviation is expected. Physically, PSI describes the shape of the peel trace (i.e., amount of noise). $CoV_{F_{ci}}$ was a statistical measure of inter-sample repeatability (sample standard deviation/average).

Fabri-Fuse showed low oscillation (SSA=0.69±0.29 N) and the lowest $CoV_{F_{ci}}(4.0\%),$ indicating the most repeatable behavior among adhesives. JB Weld showed the lowest absolute SSA (0.55±0.28 N), but a substantially higher $CoV_{F_{ci}}(22.3\%)$, indicating that peel traces with low oscillation did not guarantee the most repeatable behavior. E6000 showed the largest oscillation SSA (4.05±0.83 N) and $CoV_{F_{ci}}(14.6\%)$. Relative to E6000, the SSA values of Fabri-Fuse and JB Weld were lower by approximately 83% and 86%, respectively. Additionally, Fabri-Fuse reduced sample-to-sample variability ($CoV_{F_{ci}})$ by approximately 73% relative to E6000 and 82% relative to JB Weld. Lower sample-to-sample variability is practically important because a single average force can obscure mechanically debonding behavior [5,6]. Overall, these results show that SSA and $CoV_{F_{ci}}$ should be interpreted together to provide the most information about adhesive performance.

**Figure 4:**
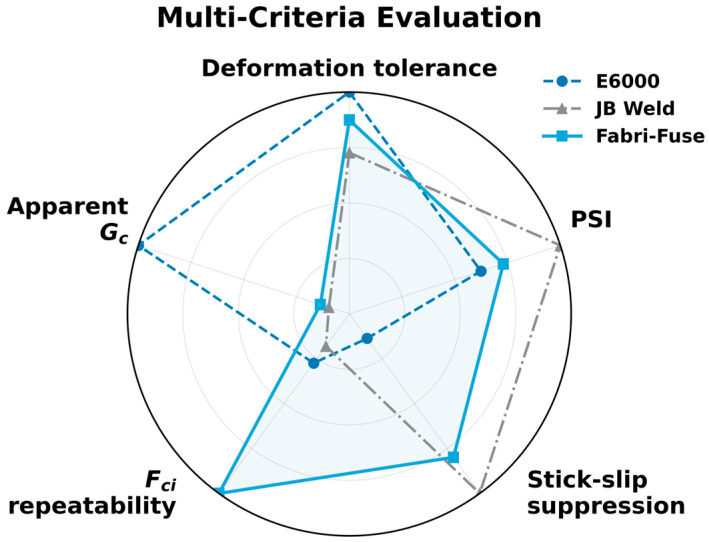
Multi-criteria evaluation of adhesives for soft, flexible substrates considering apparent fracture energy, displacement and break, peel-trace shape, stability, and repeatability. Larger values indicate better performance on each axis. The radar plot is a visualization tool for material selection rather than an intrinsic adhesive property ranking.

For practical evaluation of the adhesives, the multiple criteria were considered using a radar plot (Figure 4). The radar plot includes apparent fracture energy, normalized deformation tolerance, PSI, stick–slip suppression, and initiation repeatability. It provides a summary evaluation for selecting adhesives on fabric substrates beyond selecting the adhesive with the highest peel strength. For visualization, each criterion was converted to a normalized score so that larger values indicated more favorable performance.

Using this practical multi-criteria selection, Fabri-Fuse provided the most balanced overall performance. This result demonstrates that this multi-criteria selection considers the noise in the peel trace (PSI) and deformation at break, in addition to fracture energy. While E6000 demonstrated high fracture energy and deformation at break, it received a lower overall ranking due to larger oscillations. JB Weld ranked the highest in terms of force-trace oscillation but showed lower deformation at break and lower repeatability than Fabri-Fuse. This evaluation demonstrates a proof-of-concept of an alternative to selecting the adhesive with the highest peel strength.

### 3.4. Functional Evaluation

To evaluate the functional performance of the adhesives, we examined static shear behavior, i.e., load bearing, and the electrical continuity of a conductive path under strain. We compared the functional performance of the adhesive with the highest peel strength/fracture energy (E6000) and the highest overall performance using the multi-criteria selection (Fabri-Fuse). The load-bearing behavior of a fabric–Fabri-Fuse–fabric interface was evaluated in a shear configuration (Figure S5). Shear response depends on both cohesive and adhesive contributions and can provide information on how well the adhesive interface supports loading on the substrate, which is of practical importance for wearable devices [30]. The 82% nylon/18% spandex–Fabri-Fuse system sustained a static shear failure load of $F_{s}\;=\;80\;N$ before failure. Representative photographs of the static shear configuration and post-test specimen are provided in Figure S5. For comparison, peel force (calculated from $F_{c}/w$ and the sample width) was approximately 7.8 N. In the present system, the static shear failure load was much larger than the corresponding peel force, as expected given the different loading conditions. In comparison, the static shear load for E6000 was $F_{s}>175\;N$ at failure initiation. This lower-bound value was greater than 2.2 times higher than the result for Fabri-Fuse. This trend was consistent with the trend of peel strength for the adhesives. Peel and shear tests can be interpreted as related, but not interchangeable, measures of adhesive-joint performance because they probe the same bonded system under different stress states, edge constraints, and failure conditions [31].

The performance of Fabri-Fuse was also further examined when integrating conductive traces onto fabric substrates to determine if the peel test results translated into improved device-level performance [32]. E6000 was also examined for comparison because it showed the highest peel strength.

**Figure 5:**
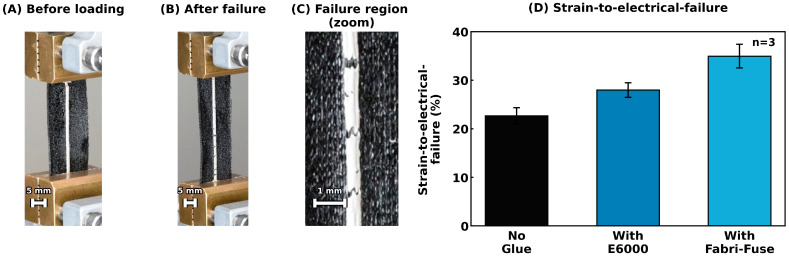
Demonstration of adhesive-dependent electromechanical performance. (**A**–**C**) Conductive trace on fabric before testing, after testing, and magnified view of the conductor region after failure. Scale bar 5 mm in (**A**), 5 mm in (**B**), and 1 mm in (**C**). (**D**) Strain-to-electrical-failure (%) for conductive textile assemblies prepared with no glue, with E6000 (highest peel strength), and with Fabri-Fuse (*n* = 3).

To mimic a wearable device, conductive traces were printed on the fabric substrate using a flexible silver conductive ink by direct ink writing (Figure 5A–C). To evaluate the role of the adhesive, three conditions were investigated: conductive traces printed on textile with no adhesive, textile with a Fabri-Fuse layer, or textile with an E6000 layer. For the sample with no adhesive, the conductive trace was printed directly onto the fabric substrate. For samples with adhesives, Fabri-Fuse or E6000 was first applied to the fabric surface, and the conductive ink was printed directly onto the uncured adhesive layer. The prepared specimens were evaluated under uniaxial extension, while current across the printed conductive path was monitored. Strain-to-electrical-failure was determined at the point where the current dropped abruptly to zero.

The no-adhesive sample exhibited a strain-to-failure of 22.66 ± 1.68% (Figure 5D). In contrast, the Fabri-Fuse-bonded assembly reached 34.95 ± 2.43% before electrical failure. In practical terms, the measured performance up to approximately 35% strain is sufficient for applications such as respiration monitoring and motion sensing [33,34]. The E6000-bonded assembly exhibited a strain-to-failure of 27.98 ± 1.47%, an improvement relative to the no-adhesive control but remaining lower than Fabri-Fuse. While E6000 had a higher peel strength, Fabri-Fuse increased the strain-to-failure by 20% compared to E6000. This comparison further supports the multi-criteria evaluation approach for adhesive selection because the adhesive with the highest peel strength did not produce the best functional outcome.

## 4. Conclusions

We evaluated fracture energy and energy dissipation of various commercial glues on a model fabric (82% nylon/18% spandex knit) substrate to probe the relative effects of the substrate and the adhesive properties. The fracture energy measured was ~1000 J/m^2^, as expected for pressure-sensitive adhesives. The plastic work of bending ($G_{d}$) was much less than the adhesive fracture energy ($G_{c}$), resulting in small correction parameters (<0.1%), which are significantly smaller than correction parameters for polymer films. Toward practical applications, we developed a multi-criteria framework for evaluating adhesives on soft, flexible textile substrates. By combining $F_{c}/w$ and $G_{c}$ with displacement at break, PSI, SSA, and $CoV_{F_{ci}}$, the framework evaluated peel-trace shape and stability in addition to conventionally calculated peel strength. Although E6000 had the highest peel strength, using multiple criteria, Fabri-Fuse was selected as the best candidate because it showed about 20% greater displacement at break than JB Weld, the lowest $CoV_{F_{ci}}$, and low stick–slip amplitude. The selected adhesive demonstrated promising performance during functional evaluation: the Fabri-Fuse-bonded assembly tolerated 34.95 ± 2.43% strain before electrical failure, compared with 22.66 ± 1.68% for the untreated fabric. Additionally, while E6000 had a higher peel strength, Fabri-Fuse increased the strain-to-failure by 20% compared to E6000. Thus, this work provides a practical and mechanics-informed basis for selecting adhesives for fabric interfaces.

## Figures and Tables

**Table 1:** Summary of peel strength ($F_{c}/w$) and selected IC-Peel output parameters ($G_{c}$, $G_{d}$, $G_{total}$, G, correction %, and $\sigma_{max_{\left(o\right)}}$) for the fabric–adhesive–fabric samples. $G_{c}$ is the apparent fracture-energy output. $G_{d}$ is the plastic bending-work contribution. $G_{total}$ is the energy value, including the stored strain energy and tensile dissipation in the peel arm. G is the total input energy. The correction value is the bending-work contribution ($G_{d}/G$) and $\sigma_{max_{\left(o\right)}}$ is the IC-Peel-calculated maximum stress in the damage zone. Values are reported as mean ± SD for n=3.

Adhesive	$F_{c}/w$ ($N\;{\mathrm{mm}}^{-1}$)	$G_{c}$ ($J\;m^{-2}$)	$G_{d}$ ($J\;m^{-2}$)	$G_{total}$ ($J\;m^{-2}$)	G ($J\;m^{-2}$)	Correction (%)	$\sigma_{max_{\left(o\right)}}$ (MPa)
E6000	1.820 ± 0.266	8673 ± 1545	3.28 ± 0.04	8677 ± 1545	3640 ± 532	0.091 ± 0.014	9.30 ± 0.82
Fabri-Fuse	0.364 ± 0.016	1188 ± 69	0	1188 ± 69	727 ± 33	N.A.	3.45 ± 0.10
JB Weld RTV silicone	0.278 ± 0.059	839 ± 237	0	839 ± 237	556 ± 118	N.A.	2.88 ± 0.40
Guo Elephant 407	N.D.	N.D.	N.D.	N.D.	N.D.	N.D.	N.D.

N.A. = not applicable; N.D. = not determined. Values for Guo Elephant 407 were not determined because no stable plateau in the peel trace was identified.

## Data Availability

The original data presented in this study are openly available from Zenodo at: https://doi.org/10.5281/zenodo.20278428. The archived dataset includes the data and supporting documentation for the fabric–adhesive–fabric T-peel force–displacement source data. The accompanying Python 3.11/Jupyter analysis workflow is available in the public GitHub repository Peel Trace Evaluation for Soft Substrates at: https://github.com/VCU-Soft-Functional-Materials-Lab/Peel-Trace-Evaluation-for-Soft-Substrates (accessed on 20 May 2026). The software record for all archived versions is available at: https://doi.org/10.5281/zenodo.20278327, and the specific v1.4.0-rc15 software release used for the manuscript baseline analysis is archived at: https://doi.org/10.5281/zenodo.20301242. The software archive also includes the built-in Scotch Magic Tape 810 validation/benchmark file used to check the force-trace extraction workflow.

## Supplementary Materials

The following supporting information can be downloaded at: https://www.mdpi.com/article/10.3390/polym18121504/s1. Figure S1: (A) Fabric–adhesive-fabric sample preparation and (B) representative T-peel test mounting. The fabric orientation was maintained to ensure the direction of peel was parallel to the wales of the fabric. This orientation was consistent for all samples. Figure S2: Representative force windows and Top5/Bottom5 extraction for (A) E6000, (B) FabriFuse, (C) JB-Weld and (D) Elephant 407. Figure S3: Images of the sample after the peel test. Figure S4: Relationship between peel strength and additional performance criteria considered. Figure S5: (A) Configuration for evaluating static shear of Fabri-Fuse-Fabric loop sample loaded vertically until adhesive debonding (B) image of sample after failure. Table S1: Summary of commercial adhesives evaluated. Table S2: Replicate-level tensile-fit outputs used to derive substrate inputs for IC-Peel. Table S3: Scotch Magic™ Tape 810 T-peel results for benchmarking results used for force-trace benchmark. Values are reported as mean ± SD. Table S4: Results from IC-Peel analysis.

## Abbreviations

The following abbreviations and mathematical symbols are used in this manuscript:

%	Percent
°C	Degrees Celsius
ASTM	ASTM International
$b_{peel}$	Peel specimen width used as an IC-Peel input
CoV	Coefficient of variation
$CoV_{F_{ci}}$	Coefficient of variation of crack-initiation force
Correction	IC-Peel correction value reported as a percentage
DIW	Direct ink writing
E	Elastic modulus of the peel arm/substrate from tensile fitting
ESIS	European Structural Integrity Society
$F_{c}$	Representative conventional peel force extracted from retained peaks and valleys
$F_{c}/w$	Width-normalized peel force, also reported as peel/bond strength
$F_{ci}$	Crack-initiation force, defined as the mean retained local peak force in the selected analysis window
$F_{p,i}$	Retained local peak force i within the selected analysis window
$F_{s}$	Static shear failure load
$F_{v,j}$	Retained local valley force j within the selected analysis window
${\bar{F}}_{win}$	Mean force within the selected analysis window
${\bar{F}}_{ci}$	Mean crack-initiation force across replicate specimens
${\bar{F}}_{p}$	Mean retained local peak force within the selected analysis window
${\bar{F}}_{v}$	Mean retained local valley force within the selected analysis window
G	Total input energy reported by IC-Peel
$G_{c}$	Apparent IC-Peel-computed adhesive fracture energy
$G_{d}$	Plastic bending-work contribution reported by IC-Peel
$G_{total}$	IC-Peel energy value after accounting for stored strain energy and tensile dissipation in the peel arm
gsm	Grams per square meter
h	Peel-arm thickness used as an IC-Peel input
I	Electrical current measured during electromechanical testing
IC-Peel	Imperial College Peel
$in\;min^{-1}$	Inch per minute
J m^−2^	Joule per square meter; unit for fracture energy or energy release rate
LDPE	Low-density polyethylene
$L_{0}$	Initial gauge length used for strain calculation
mm	Millimeter
$\mathrm{mm}\;\min^{-1}$	Millimeter per minute
$mm\;s^{-1}$	Millimeter per second
mV	Millivolt
MPa	Megapascal
N	Newton
N.A.	Not applicable
N.D.	Not determined
n	Number of replicate specimens
N mm^−1^	Newton per millimeter; unit used for width-normalized peel force or peel/bond strength
$n_{PL}$	Power-law hardening exponent from tensile constitutive fitting
PSI	Peel stability index
RH	Relative humidity
RTV	Room-temperature vulcanizing
$s_{F,win}$	Sample standard deviation of force within the selected analysis window
$s_{F_{ci}}$	Sample standard deviation of crack-initiation force across replicate specimens
SD	Standard deviation
SMU	Source-measure unit
SSA	Stick–slip amplitude
t	Peel-arm or fabric thickness used in the nominal stress calculation
$V_{app}$	Applied voltage during electromechanical testing
w	Peel specimen width used to normalize peel force
$x_{break}$	Displacement at break
ε	Engineering strain
$\varepsilon_{y}$	Yield strain from tensile constitutive fitting
θ	Peel angle used as the IC-Peel input
$\sigma_{ci}$	Nominal peel-arm axial stress at crack initiation
$\sigma_{max_{\left(o\right)}}$	IC-Peel-calculated maximum stress in the damage zone
$\sigma_{y}$	Tensile-derived substrate yield stress
