# Supplementary material for: Multi-Criteria Selection of Adhesives for Wearable Textiles
Source: Polymers (Basel). 2026 Jun 16;18(12):1504. doi: 10.3390/polym18121504 (PMC13306619; doi:10.3390/polym18121504)
Supplement: Supplementary file 1 [file polymers-18-01504-s001.zip › polymers-4362235-supplementary.pdf]

Supporting Information

Multi-Criteria Selection of Adhesives for Wearable Textiles

Bhalaji Yadav Kantepalle[1], Udena Epitawala Arachchige[2], Daeha Joung[2] and Christina Tang[1,*]

[1] Chemical and Life Science Engineering, Virginia Commonwealth University, Richmond, VA 23284, USA
[2] Department of Physics, College of Humanities and Sciences, Virginia Commonwealth University, Richmond, VA 23284, USA; epitawalaaru@vcu.edu (U.E.A.); joungd2@vcu.edu (D.J.)
* Correspondence: ctang2@vcu.edu

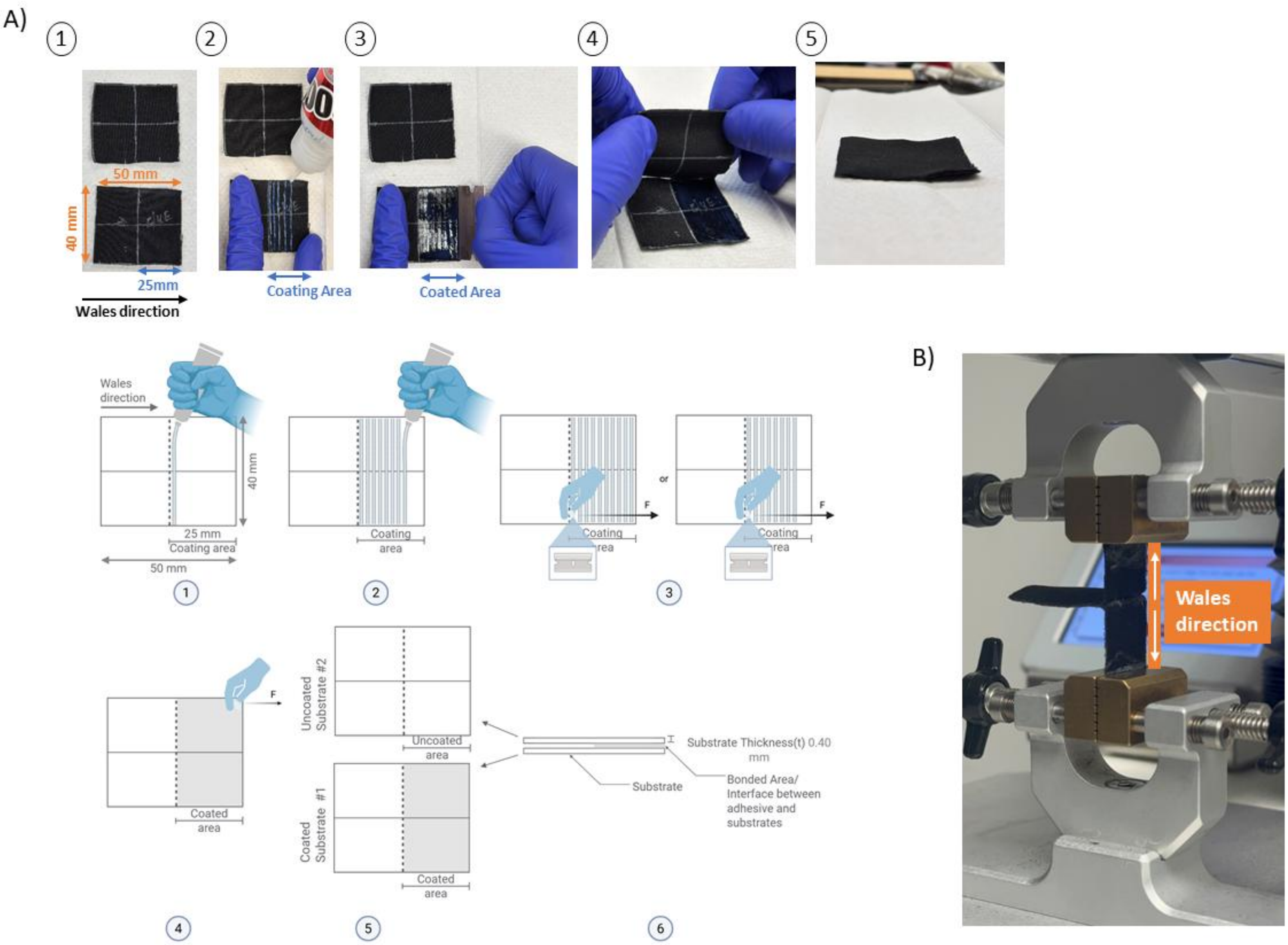

Figure S1. (A) Fabric–adhesive-fabric sample preparation and (B) representative T-peel test mounting. The fabric orientation was maintained to ensure the direction of peel was parallel to the wales of the fabric. This orientation was consistent for all samples.

Table S1. Summary of commercial adhesives evaluated

**Product**	**Adhesive class**	**Adhesive category**	**Sustained weight applied**	**Nominal curing / handling condition***
J-B Weld 31310 All-Purpose RTV Silicone	Pressure sensitive adhesive	RTV silicone	Yes	24 h,<br><br>Applied weight
E6000 565004 Fabri-Fuse Adhesive	Pressure sensitive adhesive	Rubber-based adhesive	Yes	24 h cure;<br><br>Applied weight

Eclectic Products E6000 Industrial Strength Adhesive	Pressure sensitive adhesive	Solvent-based adhesive	Yes	24 h cure;<br><br>Applied weight
Guo Elephant 407 instant adhesive	Structural adhesive	Cyanoacrylate adhesive	No	1 h

Methods S1.

T-Peel Data Analysis

Force displacement data was baseline corrected. The first 5 mm of displacement was not considered. Following the first 5 mm, a stable force window was selected by considering force data with displacement $x_i \leq x \leq x_i + 25$ mm. Candidate force windows were required to contain at least 10 data points and were not allowed to extend into the terminal 20% of the recorded displacement range. To ensure selection of a force plateau, a slope/drift criterion: $\mid m \mid L_{win}/\bar{F}_{win} \leq 0.25 \mid$, where m is the fitted force–displacement slope within the candidate force window, $L_{win} = 25\ mm$, and $\bar{F}_{win}$ is the mean force in that candidate force window was used. No mean-force cutoff was applied. Among the candidate force windows that satisfied the mean-force and minimum-data requirements, the sample standard deviation of the baseline-corrected force values within each valid window was calculated as $s_F = \sqrt{\left(\frac{\sum_{j=1}^{n} (F_j - \bar{F})^2}{n-1}\right)}$. where F_j is the baseline-corrected force at point j, $\bar{F}$ is the mean force in that candidate window, and n is the number of data points in the window. The force window with the lowest s_F was selected for further analysis (Top5/Bottom5 extraction). This procedure was applied to all adhesive systems and to the Scotch 810 benchmark without adhesive-specific adjustment.

Within the selected force window, local maxima and minima were detected directly from the unsmoothed baseline-corrected force trace. Peak detection used a prominence threshold of 0.003 times the global maximum baseline-corrected force for that trace and a minimum peak distance of one data point. Horizontal peak-span metric (spanning at least 10% of the selected window length, i.e., ≥ 2.5 mm for a 25.0 mm window) was computed to identify the 5 local minima and 5 local maxima. Representative peel traces (i.e., force vs. displacement), the selected force windows, and the Top5/Bottom 5 for the adhesives evaluated are shown in Supplementary Figure S2. No stable plateau was identified for Elephant 407 and no further analysis was performed.

Displacement at break was determined by scanning the baseline-corrected force trace after the selected stable analysis window. Displacement at break was defined as the first post-window displacement point at which the force decreased to less than or equal to $0.10F_c$, where F_c is the stable-window peel force.

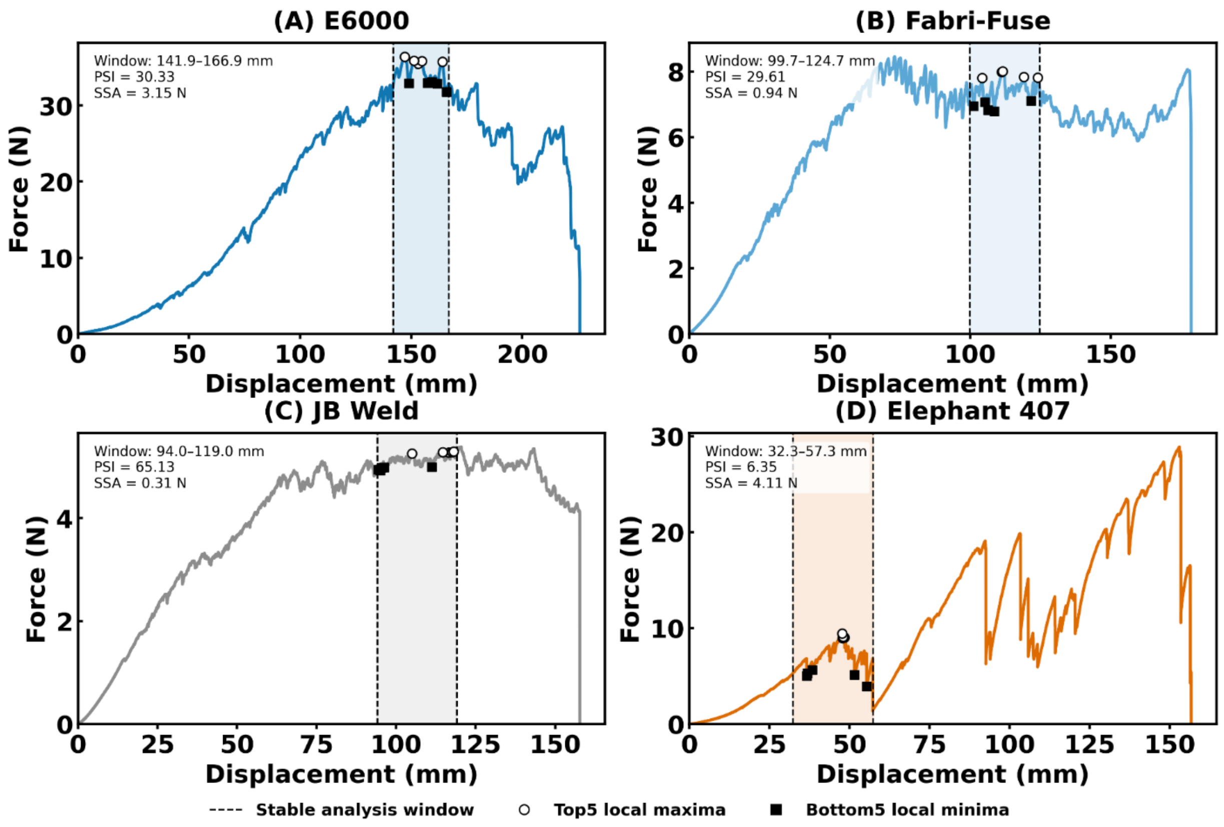

Figure S2. Representative force windows and Top5/Bottom5 extraction for (A) E6000, (B) FabriFuse, (C) JB-Weld) and (D) Elephant 407.

IC-Peel Analysis

For analysis of fracture energy and energy dissipation, IC-peel analysis was performed. Tensile force–displacement data of the fabric substrate were converted to engineering strain and nominal stress using $\varepsilon = \Delta L/L_0$ and $\sigma = F/(b_{tensile} \times h)$, where L_0 is the effective gauge length, $b_{tensile}$ is the tensile specimen width, and h is the fabric thickness. Toe/slack was removed setting the first run of five consecutive points above a 1.0 N force to a displacement of zero. The elastic modulus E of the substrate was estimated from the $0.02 - 0.08$ strain window using a slope-through-origin fit. The post-knee response was fit for $\varepsilon \geq 0.06$ using power-law and bilinear constitutive models. These two constitutive forms were selected because bilinear and power-law stress–strain descriptions are used in established IC-Peel/flexible-laminate peel protocols to represent peel-arm behavior beyond the initial elastic regime [S1]. During fitting, yield strain $\varepsilon_y = \sigma_y/E$ was constrained to lie within the fitted strain. The better-fitting model for each specimen was selected based on R^2 and RMSE. The resulting substrate inputs, including $E, \sigma_y, \varepsilon_y$, and the power-law hardening exponent n_{PL}, were exported for IC-Peel analysis.

For the power-law model, the elastic region was described by $\sigma = E\varepsilon$ for $\varepsilon \leq \varepsilon_y$, and the post-yield region was described by $\sigma = K \times \varepsilon^{n_{PL}}$, where $\varepsilon_y = \sigma_y/E$. The coefficient K was not independently fitted; $K = \sigma_y/(\varepsilon_y^{n_{PL}})$ to enforce continuity at yield. For the bilinear model, the elastic region was described by $\sigma = E \times \varepsilon$, and the post-yield region was described by $\sigma = \sigma_y + E_p \times (\varepsilon - \varepsilon_y)$, where $E_p = \alpha \times E$. The

power-law fit better fit the strain-stiffening response of the knitted fabric in the tested direction. Because the power-law model provided the better fit for all three tensile specimens, the average power-law parameters for each sample were used for the primary IC-Peel calculations. Bilinear parameters are reported for comparison.

For IC-Peel calculations, the fabric-adhesive-fabric peel width b_{peel}, peel-arm thickness h, peel angle θ, *and* adhesive-specific force F_c were combined with the tensile-derived substrate inputs using the Imperial College IC-Peel 2006 software [S1]. The tensile specimen width $b_{tensile} = 50$ was used only to convert tensile force to nominal tensile stress, whereas the peel specimen width $b_{peel} = 20$ mm was used in the IC-Peel calculation.

In the IC-Peel input table, $\theta = 180°$ was used as the T-peel-equivalent high-angle work-term input. This assignment does not indicate that a separate 180° peel experiment was performed, nor does it treat the T-peel test as two independent 90° peel tests. Rather, in the ideal flexible-arm limit, symmetric T-peel and 180° peel have the same high-angle external-work factor because both peel arms move away from the crack front during debonding. In the peel-energy expression, this geometry enters through the term $(1 - cos\ \theta)$, which equals 2 when $\theta = 180°$ and 1 when $\theta = 90°$ [S1] [S2]. Therefore, θ=180° was used as the appropriate high-angle representation of the T-peel geometry in the IC-Peel calculation. For the present the fabric-adhesive-fabric samples, this geometric term was not used alone; peel-arm extensibility and deformation were accounted for through the tensile-derived substrate inputs and the IC-Peel regime classification [S1], [S2].

The IC-Peel calculation was therefore interpreted using the nonlinear-extension and bending-correction, where the energy estimate includes the peel-angle work term, the tensile stress–strain contribution of the peel arm, and, when applicable, the bending-dissipation correction. Accordingly, the reported G_c values are treated as configuration-dependent, configuration-dependent apparent fracture-energy estimates than intrinsic adhesive material properties [S1], [S2].

Notably, the model captures the measured nonlinear extension of the fabric strip, including the post-knee strain-stiffening behavior, but it does not explicitly resolve yarn-level loop rotation, yarn–yarn contact, through-thickness compression, local adhesive penetration, or spatially varying crack-front interaction with the knit architecture.

Table S2. Replicate-level tensile-fit outputs used to derive substrate inputs for IC-Peel.

**Parameter**	**Unit**	**Specimen 1**	**Specimen 2**	**Specimen 3**	**Mean ± SD**
Tensile specimen width, $b_{tensile}$	mm	50	50	50	50.0 ± 0.0
Thickness, h	mm	0.40	0.40	0.40	0.40 ± 0.0
Elastic modulus, E	GPa	0.000282	0.000359	0.000405	0.000349 ± 0.000060
Yield stress, σ_y	MPa	0.389	0.705	1.074	0.723 ± 0.343
Yield strain, ε_y	%	137.8	196.3	265.3	199.8 ± 63.8
Power-law exponent, n_{PL}	—	2.762	3.063	3.411	3.079 ± 0.325
Power-law R^2	—	0.993	0.999	0.998	0.997 ± 0.003
Power-law RMSE	MPa	0.472	0.089	0.256	0.272 ± 0.192
Bilinear parameter, α	—	0.999	0.999	0.999	0.999 ± 0.000
Bilinear R^2	—	-0.540	-0.196	-0.321	-0.352 ± 0.174
Bilinear RMSE	MPa	7.077	3.533	6.813	5.808 ± 1.975

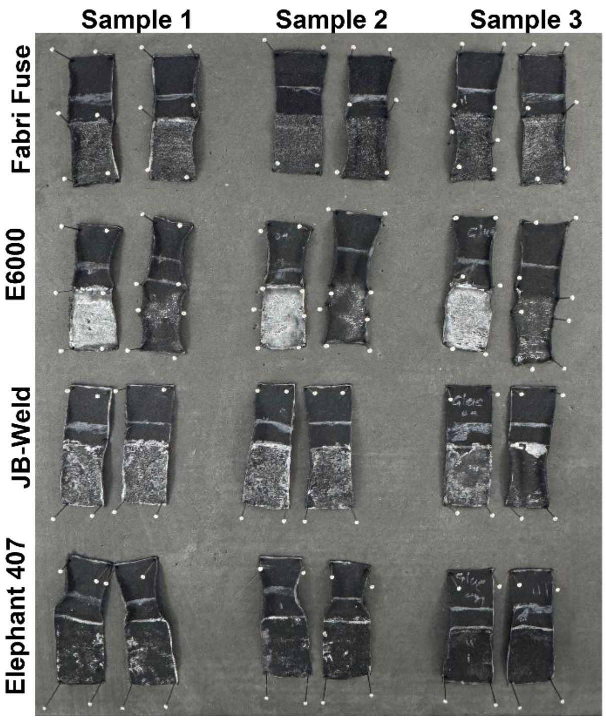

Figure S3. Images of the sample after the peel test.

Analysis of Scotch Magic™ Tape 810

Scotch Magic™ Tape 810 was used as a benchmark to confirm that the selected peel-window logic and Top5/Bottom5 force-extraction procedure produced reasonable and repeatable outputs on a uniform reference material.

For the Scotch Magic™ Tape 810-to-Scotch Magic™ Tape 810 T-peel benchmark, tape strips with a nominal width of 19 mm and total thickness of approximately 0.060 mm were cut to a length of 50 mm. Two tape strips were aligned in the same direction to form an overlap region of approximately 19 mm × 25 mm and pressed manually to establish full contact. A temporary non-adhesive paper gripping strip was applied only to the free tape arms to prevent unintended adhesion to the grips during clamping. This temporary gripping strip was outside the bonded analysis region and was not treated as part of the bonded tape–tape interface. These specimens were tested using a $20\,mm$ initial clamp gap, and a crosshead speed of $10\,mm\,min^{-1}$ ($0.17\,mm\,s^{-1}$) on a texture analyzer (TA.XTPlus Connect).

The Scotch Magic™ Tape 810-to-Scotch Magic™ Tape 810 T-peel benchmark produced $F_c/w\ =\ 0.187\ \pm$ $0.024\,N\,mm^{-1}$ at a crosshead speed of $0.17\,mm\,s^{-1}$. This result was comparable to the Scotch 810-to-Scotch 810 adhesive-layer benchmark reported by Gilman et al. ($0.198\ \pm\ 0.005\,N\,mm^{-1}$, converted from $198\ \pm$ $5\,N\,m^{-1}$), measured using $19\,mm$ Scotch 810 tape in T-peel at $100\,mm\,min^{-1}$ ($1.67\,mm\,s^{-1}$) [S3] and confirm the efficacy of the analysis method used.

Table S3. Scotch Magic™ Tape 810 T-peel results for benchmarking results used for force-trace benchmark. Values are reported as mean ± SD.

Benchmark condition	Selected specimens	(F_c/w) $[N\ mm^{-1}]$	(F_{ci}/w) $[N\ mm^{-1}]$	Window CoV [%]	SSA [N]	PSI	Displacement at break [mm]
Present Scotch 810-to-Scotch 810 T-peel	$n_{rep} = 3$	0.187± 0.024	0.202 ± 0.025	6.28 ± 1.04	0.692 ± 0.028	16.2 ± 2.8	55.707 ± 3.814
Gilman et al. Scotch 810-to-Scotch 810 T-peel reference [S3]	Literature benchmark;20 runs reported	0.198 ± 0.005	—	—	—	—	

Table S4. Results from IC-Peel analysis. F_c is the representative peel force, and F_c/w is the corresponding peel strength. In the IC-Peel calculation, the E, σ_y, and n_{PL} are the properties of the fabric substrate determined from tensile testing. The h is the fabric thickness, b_{peel} is the sample width, and θ is the 180°. G_c is the computed apparent critical peel energy, G_d is the plastic work in bending, G_{tot} is the input energy including the stored strain energy and tensile dissipation in the peel arm and G is the total input energy; correction is $\frac{G_d}{G}$(%). $\sigma_{\max(o)}$ is the maximum stress for the damage zone.

	IC-Peel (2006) Input								IC-Peel (2006) Output						
**Adhesive**	F_c [N]	F_c/w [$N\ mm^{-1}$]	**Fabric** E [GPa]	**Fabric** σ_y [MPa]	**Fabric** n_{PL}	**Fabric h [mm]**	**T-Peel arm width** b_{peel} **[mm]**	θ [°]	**IC-Peel case**	G_c [$J\ m^{-2}$]	G_d [$J\ m^{-2}$]	G_{tot} [$J\ m^{-2}$]	G [$J\ m^{-2}$]	**Correction [%]**	$\sigma_{\max(o)}$ [MPa]
E6000	34.307	1.715	0.000349	0.723	3.079	0.4	20	180	3	8056.9	3.307	8060.2	3430.697	0.096	8.989
E6000	42.454	2.123	0.000349	0.723	3.079	0.4	20	180	3	10431.5	3.231	10434.7	4245.434	0.076	10.228
E6000	32.452	1.623	0.000349	0.723	3.079	0.4	20	180	3	7532.0	3.299	7535.3	3245.239	0.102	8.691
Fabri-Fuse	7.416	0.371	0.000349	0.723	3.079	0.4	20	180	1	1217.6	0	1217.6	741.6419	—	3.494
Fabri-Fuse	7.505	0.375	0.000349	0.723	3.079	0.4	20	180	1	1236.3	0	1236.3	750.4532	—	3.521
Fabri-Fuse	6.901	0.345	0.000349	0.723	3.079	0.4	20	180	1	1108.8	0	1108.8	690.0788	—	3.335
JB Weld	4.666	0.233	0.000349	0.723	3.079	0.4	20	180	1	661.5	0	661.5	466.5782	—	2.576
JB Weld	5.121	0.256	0.000349	0.723	3.079	0.4	20	180	1	747.0	0	747.0	512.129	—	2.737
JB Weld	6.897	0.345	0.000349	0.723	3.079	0.4	20	180	1	1108.1	0	1108.1	689.7333	—	3.334

The IC-Peel output classified E6000 in the elastic–plastic loading/unloading regime (IC-Peel case 3), whereas Fabri-Fuse and JB Weld remained in the purely elastic regime (IC-Peel case 1).

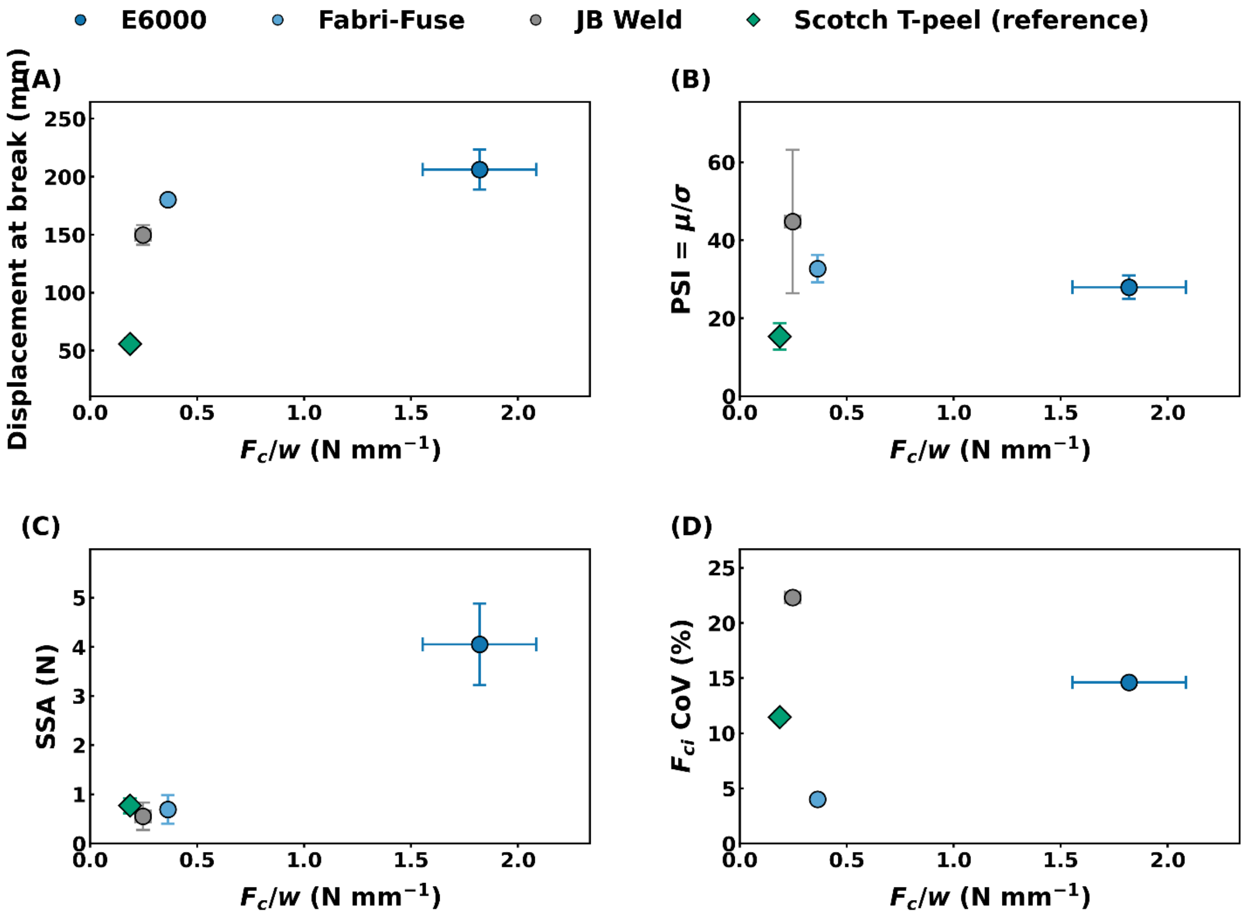

Figure S4. Relationship between peel strength and additional performance criteria considered. (A) displacement at break vs peel strength, (B) Peel Stability Index (PSI) vs. peel strength, (C) stick–slip amplitude (SSA) vs. peel strength, and (D) $CoV_{F_{ci}}$ versus peel strength (F_c/w) Points represent mean values (n = 3) and error bars indicate standard deviation where applicable. Scotch 810 was included as a reference.

A 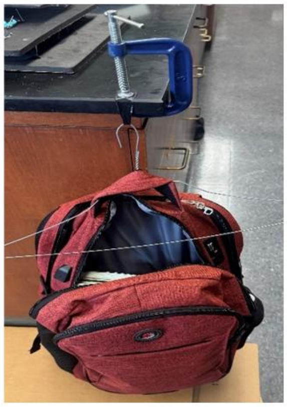

B 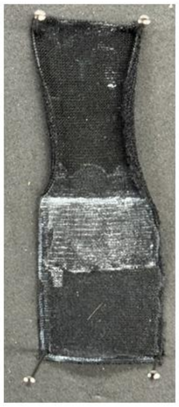

Figure S5: (A) Configuration for evaluating static shear of Fabri-Fuse-Fabric loop sample loaded vertically until adhesive debonding (B) image of sample after failure.

References:

[S1] Moore, D.R.; Williams, J.G. A Protocol For Determination Of The Adhesive Fracture Toughness Of Flexible Laminates By Peel Testing: Fixed Arm And T-Peel Methods An ESIS Protocol Revised. 2007. Available Online: https://www.imperial.ac.uk/media/imperial-college/research-centres-and-groups/adhesion-and-adhesives-group/ESIS-peel-protocol-(June-07)-revised-Nov-2010.pdf (accessed on 10 May 2026).

[S2] Bartlett, M.D.; Case, S.W.; Kinloch, A.J.; Dillard, D.A. Peel tests for quantifying adhesion and toughness: A review. *Prog. Mater. Sci.* **2023**, *137*, 101086. DOI: 10.1016/j.pmatsci.2023.101086.

[S3] A. Gilman, M. Piskarev, and M. Y. Yablokov, "Adhesive properties of PTFE modified by DC discharge," *Journal of Physics: Conference Series*, vol. 516, no. 1, Art. no. 012012, 2014, doi: 10.1088/1742-6596/516/1/012012.
